# Annotated Peruvian banknote dataset for currency recognition and classification

**DOI:** 10.1016/j.dib.2023.109715

**Published:** 2023-10-24

**Authors:** Nicolás Esleyder Caytuiro-Silva, Jackeline Melady Peña-Alejandro, Eveling Gloria Castro-Gutierrez, Jose Sulla-Torres, Benjamin Maraza-Quispe

**Affiliations:** a Universidad Católica de Santa María, Arequipa Peru; b Universidad Nacional de San Agustín de Arequipa, Arequipa Peru

**Keywords:** Banknote recognition, Peruvian banknote, Machine learning, Annotated dataset

## Abstract

The real-time detection of multinational banknotes remains an ongoing research challenge within the academic community. Numerous studies have been conducted to address the need for rapid and accurate banknote recognition, counterfeit detection, and identification of damaged banknotes [1], [2], [3]. State-of-the-art techniques, such as machine learning (ML) and deep learning (DL), have supplanted traditional digital image processing methods in banknote recognition and classification. However, the success of ML or DL projects critically hinges on the size and comprehensiveness of the datasets employed. Existing datasets suffer from several limitations. Firstly, there is a notable absence of a Peruvian banknote dataset suitable for training ML or DL models. Second, the lack of annotated data with specific labels and metadata for Peruvian currency hinders the development of effective supervised learning models for banknote recognition and classification. Lastly, datasets from different regions may not align with the unique characteristics, design, and security features of Peruvian banknotes, limiting the accuracy and applicability of models in a Peruvian context [4] To address these limitations, we have meticulously curated a comprehensive dataset comprising a total of 9,315 images of Peruvian banknotes, encompassing both old and new denominations from 2011 (old) and 2019 (new) [5]. The Peruvian banknote dataset includes denominations of 10, 20, 50, and 100 Peruvian soles. Importantly, as indicated by [5], both the 2011 and 2019 families of banknotes are currently in circulation, further enhancing the dataset's relevance for real-world applications in currency recognition and verification.

This dataset serves as a vital resource for addressing the challenges in real-time multinational banknote detection. By offering a comprehensive collection of images of Peruvian banknotes, both old and new, this dataset fills a critical gap in the field of banknote recognition. Researchers can utilize it to train and evaluate advanced machine learning and deep learning models, ultimately enhancing the accuracy of banknote processing systems.

Specifications Table

**Table:** 

Subject	Machine Learning
Specific subject area	Banknote detection and identification
Data format	Raw Labelled
Type of data	The Peruvian Banknote dataset comprises .jpg images with dimensions of 640 × 480 pixels and a resolution of 72 DPI.
Data collection	The images of Peruvian banknotes were captured using a high-resolution smartphone camera. The original banknote images in .jpg format have dimensions of 3072 × 3072 pixels. These images were resized to 640 × 480 pixels. There are a total of 16 classes of Peruvian banknotes, including denominations of 10, 20, 50, and 100 Peruvian soles (considered as separate classes for both the obverse and reverse sides of the banknotes). The banknote images were taken under various environmental conditions, including dark backgrounds, illuminated backgrounds, cluttered environments, and with folded banknotes, both within and outside the laboratory.
Data source location	Catholic University of Santa Maria De Arequipa Urb. San José, San Jose s/n, Yanahuara Arequipa, Peru
Data accessibility	Repository name: Peruvian Banknotes [6] Data identification number(doi): 10.17632/22jmt8xhpn.1 Direct URL to data: https://data.mendeley.com/datasets/22jmt8xhpn/1

## 1 Value of the Data

• This dataset represents a significant and valuable addition to the available resources on the internet, comprising a total of 9,315 high-quality images spanning 16 distinct categories.
• Notably, it includes both old and new denominations of Peruvian banknotes, offering comprehensive coverage of both the obverse and reverse sides of the bills.
• The dataset serves as a highly valuable resource for the development of applications centered around the classification and detection of Peruvian banknotes, making it particularly advantageous for researchers specializing in banknote classification and identification.
• Its versatility is a key asset, as it can be effectively employed for various applications, encompassing training, validation, and testing phases for both banknote classification and identification models.
• Moreover, the dataset plays a pivotal role in the precise valuation of Peruvian banknotes, significantly aiding financial transactions and monetary activities.
• It's important to note that this dataset is not intended for the classification of whether banknotes are genuine or counterfeit. Instead, its primary focus lies in enabling advanced machine and deep learning models for the accurate recognition and categorization of Peruvian banknotes.
• Beyond its financial applications, this dataset extends its impact to the development of tools for banknote recognition and classification, thereby benefiting not only visually impaired individuals but also bank customers and governmental agencies alike. The dataset's potential to enhance accessibility and facilitate monetary interactions underscores its broader societal significance.

## 2 Data Description

The creation of a banknote dataset is of paramount importance for several compelling reasons. Firstly, the accurate recognition of banknotes is an essential task for automated teller machines (ATMs) and currency identification machines [7] cited by [4]. Moreover, the development of systems capable of authenticating banknotes is essential [8]. Additionally, banknote recognition remains a significant challenge for individuals with visual impairments, underscoring its vital role in promoting financial accessibility [9].

**Fig. 1:**
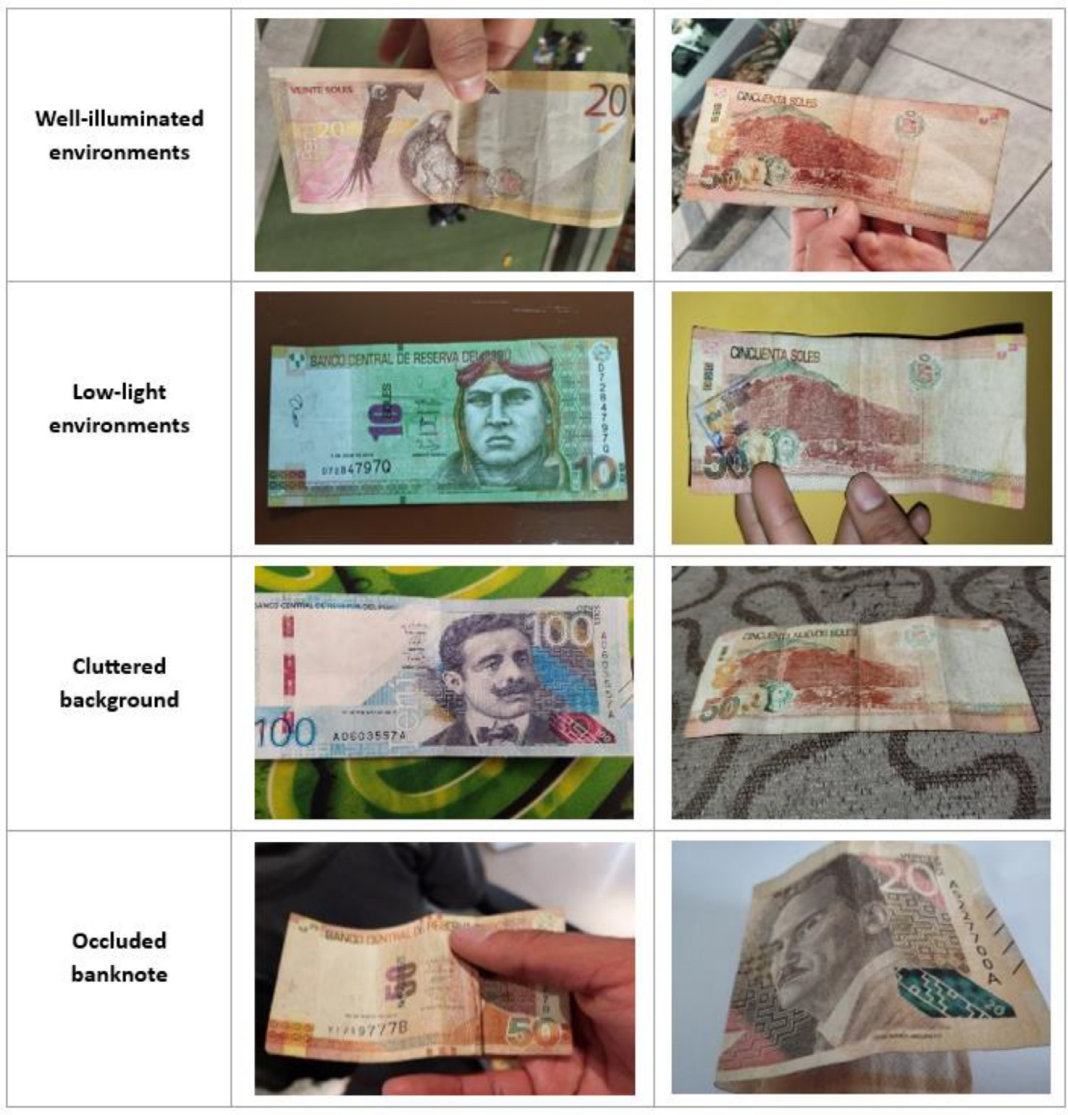
Banknote images taken in various environments (Scenario 1-4/From 7).

**Fig. 2:**
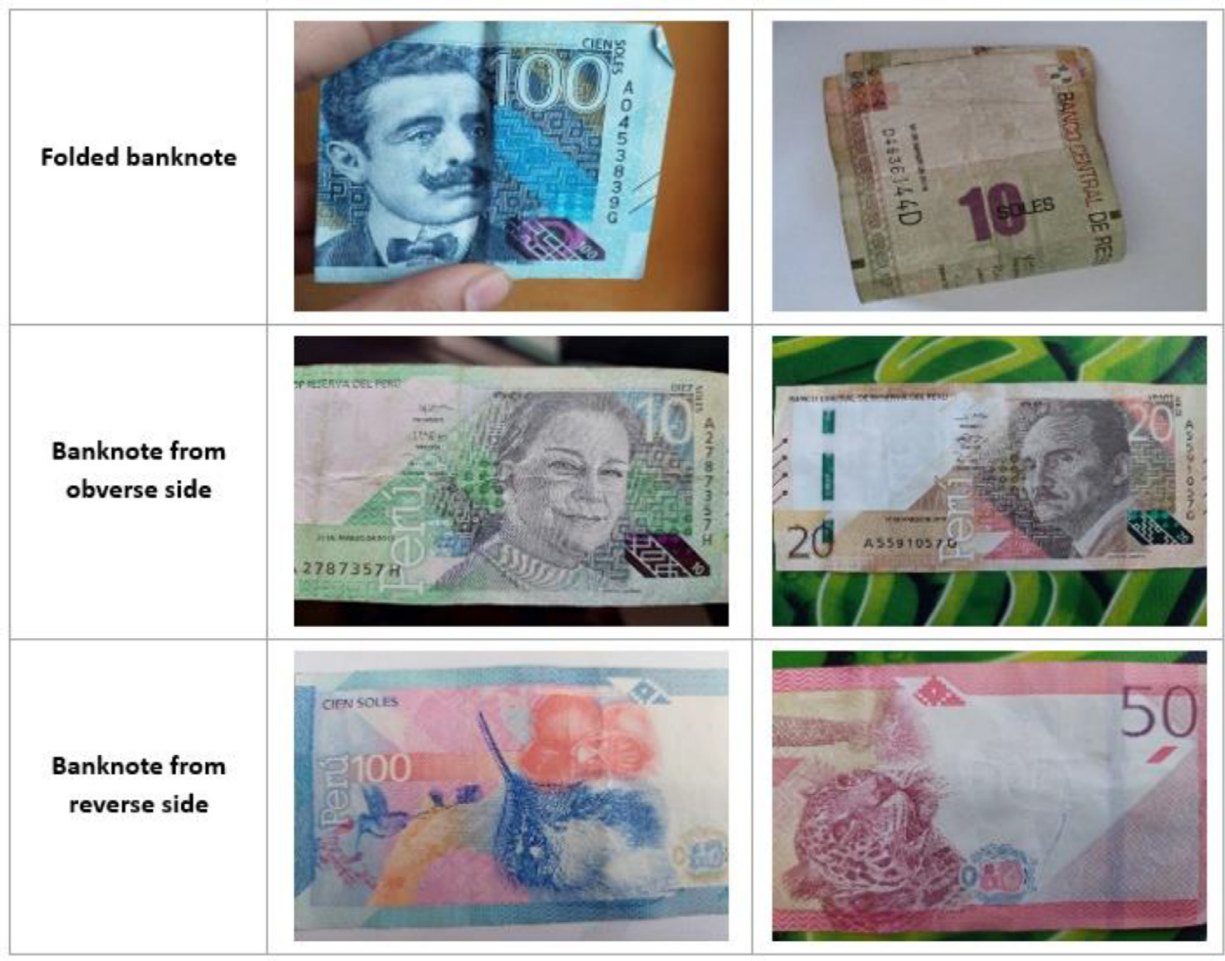
Banknote images taken in various environments (Scenario 5-7/From 7).

Our banknote dataset represents a comprehensive collection of Peruvian currency. It encompasses 16 distinct classes, encompassing denominations of 10, 20, 50, and 100 Peruvian soles. Notably, each denomination is treated as a separate class, accounting for both the obverse and reverse sides of the banknotes. The dataset includes banknote images captured under various environmental conditions, ranging from well-illuminated settings to low-light environments and cluttered backgrounds. Furthermore, it contains images of partially folded or partially obscured banknotes. Sample images from the dataset, captured in diverse environmental scenarios, are depicted in Figs. 1 and 2.

The Peruvian banknote images are stored within the “Peruvian_Banknotes” folder, serving as the central repository. This primary folder is further subdivided into three subfolders: “training_set,” “validation_set,” and “testing_set”. Both the training and validation sets comprise 16 subfolders, each housing classes representative of the dataset. These classes encompass banknotes from the 2011 and 2019 currency families [5], encompassing denominations of 10, 20, 50, and 100 Peruvian soles, and encompassing both the obverse and reverse sides of the banknotes. Finally, the testing folder includes 261 images sourced from everyday environments, summing up to a total of 9,315 images of Peruvian banknotes.

**Fig. 3:**
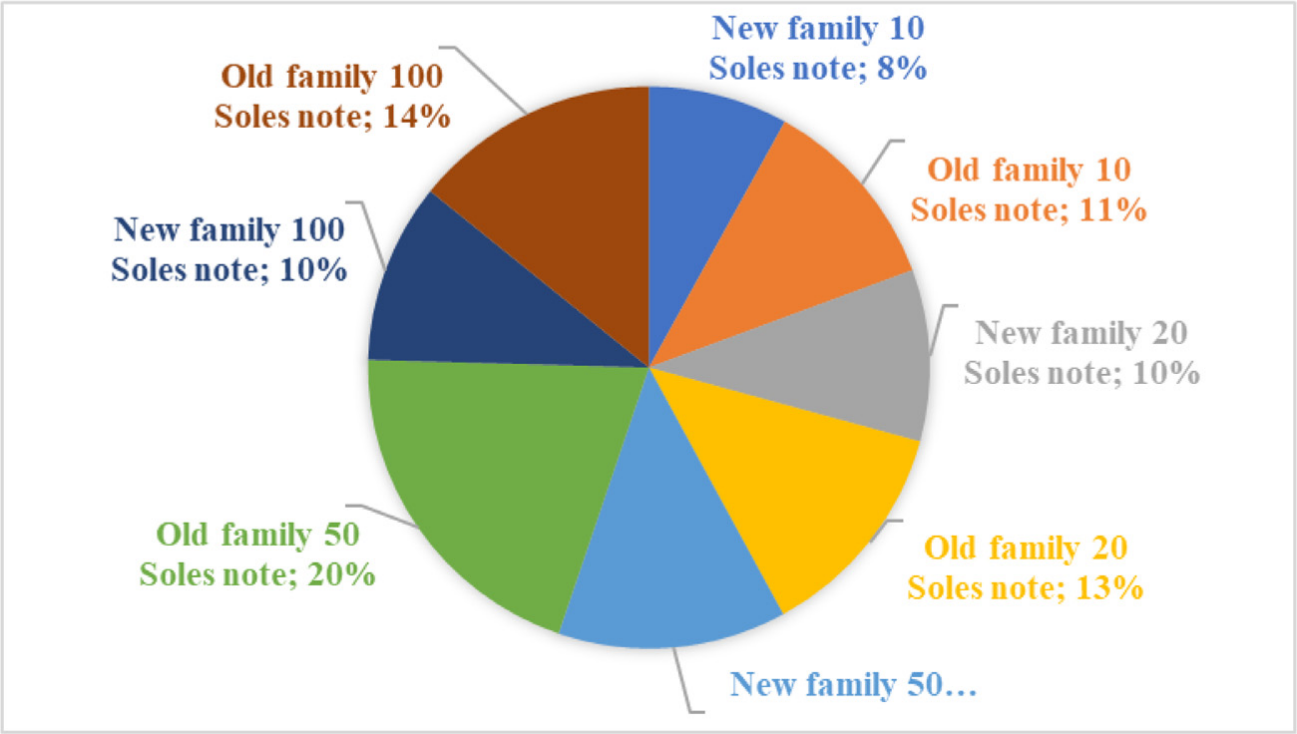
Percentage of each currency denomination in the Peruvian Banknotes dataset.

According with Table 1, the distribution of denominations in the dataset is illustrated in Fig. 3. Fig. 3 displays the representation of different banknote denominations. The 10 Soles note from the new family comprises 8% of the dataset, while the 10 Soles note from the old family accounts for 11%. The new family 20 Soles note is 10% of the dataset, while the old family 20 Soles note represents 12%. The new family 50 Soles note constitutes 13% of the dataset, while the old family 50 Soles note holds the highest share at 20%. The new family 100 Soles note is 10% of the dataset, while the old family 100 Soles note is 14%.

**Table 1:** Total denominations.

	Denomination				Total
	10	20	50	100	
Banknote family new	728	894	1190	939	3751
Banknote family old	1025	1162	1831	1285	5303
Total	1753	2056	3021	2224	9054

**Fig. 4:**
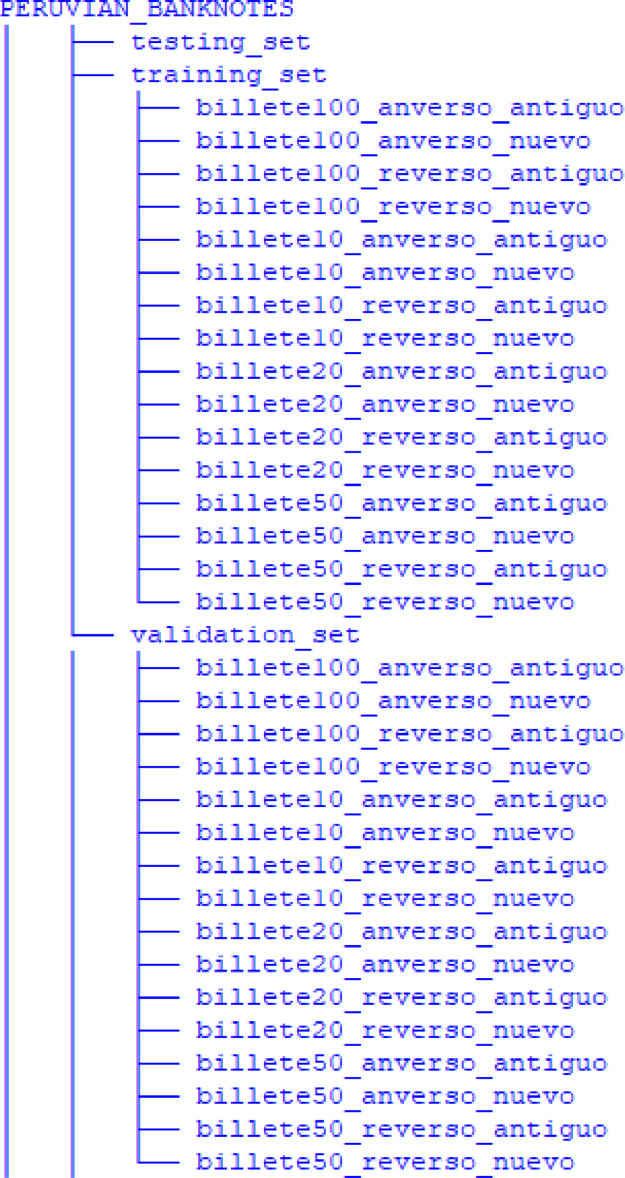
Peruvian Currency dataset directory structure.

The directory structure of the Peruvian Banknotes dataset is shown in Fig. 4. Fig. 4 describes the folder structure of the Peruvian Banknotes Dataset. The dataset's root folder, labeled as “PERUVIAN_BANKNOTES”, encompasses three primary subfolders: “training_set”, “validation_set” and “testing_set”. Within these subfolders, the dataset is further categorized into 16 distinct classes, each representing a different denomination of Peruvian currency, encompassing both the obverse and reverse sides of the banknotes.

To further clarify, this dataset exclusively focuses on genuine Peruvian banknotes for recognition and classification purposes. It does not include any counterfeit banknotes.

## 3 Experimental Design, Materials and Methods

### 3.1 Experimental design

**Fig. 5:**
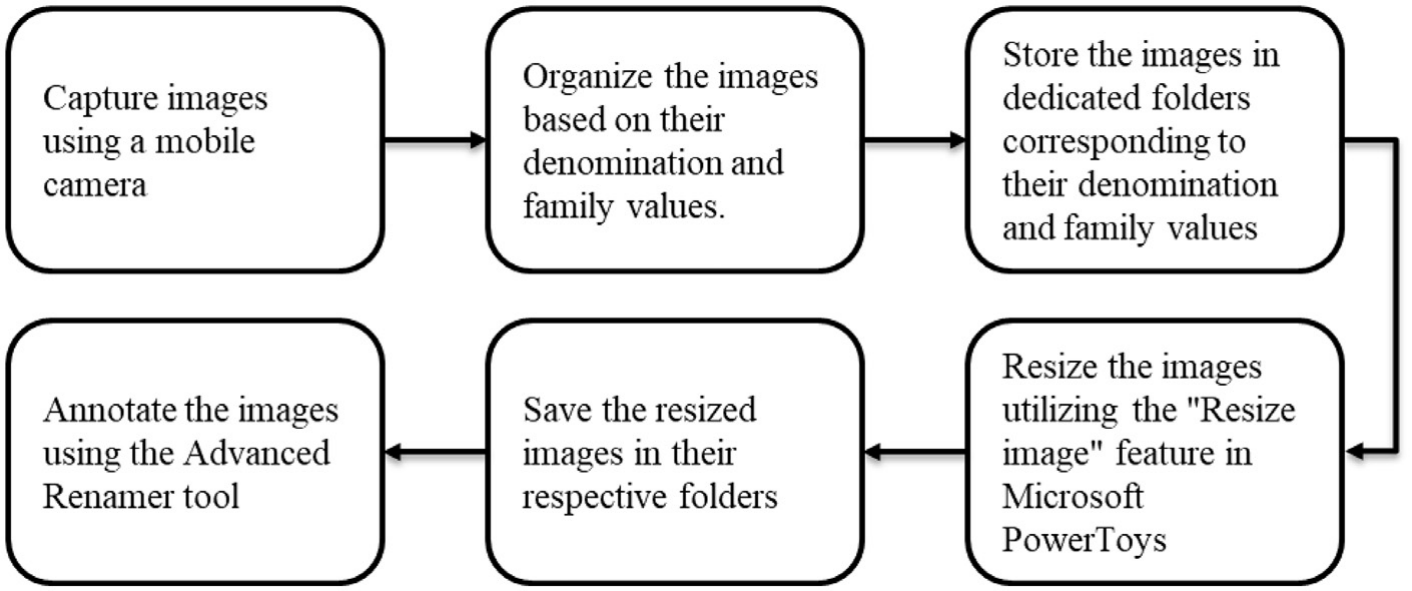
Peruvian Banknote dataset acquisition process.

The process of acquiring image data is illustrated in Fig. 5. Banknote images were obtained using the high-resolution rear camera of a Redmi Note 12 smartphone (see Table 2 for device specifications). A total of 9,315 images were captured and subsequently categorized and stored in respective folders based on their denominations and family values.

**Table 2:** Device specifications for banknote capture.

	Description
Processor	Snapdragon 685
RAM	8 GB LPDDR4X
Storage	128 GB
Front Camera	13 megapixels f/2.45
Main Rear Camera	50 MP f/1.8
Wide-angle	8 MP f/2.2
Macro	2 MP f/2.4
Operating System	Android 12 + MIUI 14

Table 3 outlines the steps involved in the data acquisition process, while Table 4 provides specifics regarding image acquisition and preprocessing tools. As indicated in Table 5, the banknote images were captured from various angles and backgrounds. Subsequently, following the capture of the Peruvian banknote images, they were meticulously organized into dedicated folders. For a comprehensive breakdown of the folder structure and file names, please refer to Table 6.

**Table 3:** Data acquisition steps.

Step	Duration	Activity
Data Gathering	April to July	Daily during daytime captured the banknote images
Image manual processing	July to September	Separate the images into their respective folders. Resize the images and change their names accordingly.

**Table 4:** Specification of image acquisition and preprocessing tools.

No.	Particular	Details
1	Camera	(i) Make and Model: Redmi Note 12 (ii) Rear Camera 50-megapixel (f/1.8) (iii) Rear autofocus
2	Battery	5.000 mAh
3	Labelling Software	Advanced Renamer
4	Resizing Software	Microsoft PowerToys
5	Image Resolution	640 × 480 pixels
6	Image Format	JPG

**Table 5:** Banknote image capture conditions.

Denominations	Direction of image Capturing	Different backgrounds considered for image capturing	No. of images of each denomination	Total No. of images
10 New and Old, 20 New and Old, 50 New and Old, 100 New and Old Peruvian Soles. Both obverse and reverse side.	Front Direction, Front Direction Rotated 180°, Backward Direction, Backward Direction Rotated 180°, Half folded.	Illuminated, Dark, cluttered, occluded.	10 Soles – 1753 20 Soles – 2056 50 Soles – 3021 100 Soles – 2224 Test Soles – 261	9315

**Table 6:** Structure and naming of image directories.

Type	Description	Nomenclature used
Directories	The captured images were organized into 16 distinct folders. These folders represent different combinations of banknote denominations, sides (obverse, reverse), and banknote families (new/old).	For example: • banknote10_obverse_old • banknote10_reverse_old • banknote10_obverse_new • banknote10_reverse_new
Files	Files Each image was labeled following specific nomenclature to enable identification and categorization during the training process. The nomenclature includes: • The dataset type to which the image belongs (training, validation, or testing). • The denomination of the banknote (10, 20, 50, or 100). • The banknote family (new or old). • A sequential enumeration for unique identification of each image.	Ejemplo de etiqueta: “training_10_old_001”

The dataset creation process lasted for 5 months, starting in April 2023, and concluding in September 2023. It is important to mention that it was a rigorous process, beginning with the capture of banknote images and involving manual image processing. The manual processing helped in reducing the image sizes and sorting each image into its appropriate folder. A total of 16 folders were used, which represent the classes for training machine and deep learning models.

### 3.2 Materials or specification of image acquisition system

The banknote images were captured using a Redmi Note 12 smartphone equipped with a 50 MP rear camera. To ensure uniformity, all original images, originally sized at 3072 × 3072 pixels, were resized to 640 × 480 dimensions using the “Resize image” utility in Microsoft PowerToys. These images were saved in .jpg format. The image capture process encompassed various environmental conditions, including different lighting conditions, backgrounds, angles, as well as scenarios involving folded and occluded banknotes, mirroring real-world situations.

Subsequently, the captured images were meticulously organized into training, validation, and testing sets. The dataset comprises a total of 16 distinct classes, representing Peruvian banknotes with denominations of 10, 20, 50, and 100 Peruvian soles. Notably, each denomination is treated as a separate class, accounting for both the obverse and reverse sides of the banknotes. For a detailed overview of the directory structure of the image dataset, please refer to Table 6. To enhance their utility, the images were annotated using the Advanced Renamer tool, with annotations and banknote images stored in their respective folders.

### 3.3 Method

All banknote images were captured using the rear camera of a Redmi Note 12 mobile phone, encompassing various angles and backgrounds. These images, originally sized at 3072 × 3072 pixels, were subsequently resized to 640 × 480 pixels using the “Resize image” utility within PowerToys. The resizing and renaming process is detailed in Table 4 for reference. Table 5 provides a comprehensive overview of the classes, the quantity of images taken, and the environmental conditions under which these images were taken.

## Limitations

None.

## Ethics statement

There is no funding present for present effort. There is no conflict of interest. The data is available in public domain.

## Ethical Statement

We appreciate the opportunity to submit our dataset, the “Annotated Dataset of Peruvian Banknotes,” for your review and possible publication in Data in Brief. We acknowledge the importance of ethics in scientific publishing and wish to assure you that we have adhered to the highest ethical standards at every stage of our work.

In accordance with the ethical publishing guidelines provided by Elsevier and Data in Brief, we have considered and complied with the following key ethical aspects:

1. **Authorship of the paper**: We have limited authorship of our article to those who have made significant contributions to the conception, design, execution, and/or interpretation of our study. Furthermore, we have ensured transparency of author contributions by including a CRediT statement in our article.
2. **Originality and plagiarism**: All materials presented in our dataset are entirely original. We have ensured that, in the event of using the work and/or words of others, it has been appropriately cited and referenced.
3. **Data access and retention**: We are prepared to provide public access to raw data related to our dataset for editorial review. We believe data availability is essential for transparency and the reliability of scientific research.
4. **Multiple, redundant, or simultaneous publication**: We are committed to not publishing manuscripts describing essentially identical research in more than one journal or primary publication. We acknowledge and comply with Elsevier's policies on prior publication.
5. **Acknowledgment of sources**: Proper acknowledgment has been given to the work of other researchers and authors whose materials have influenced our dataset.
6. **Disclosure and conflicts of interest**: Full disclosures of all relationships that could be viewed as presenting a potential conflict of interest in relation to our dataset have been provided.
7. **Fundamental errors in published works**: If we were to discover a significant error in our dataset in the future, we undertake to promptly notify the journal editor and cooperate with the retraction or correction process as necessary.
8. **Reporting standards**: An accurate and objective description of the work performed in creating our dataset, as well as a discussion of its significance, has been presented.
9. **Hazards and human or animal subjects**: No materials, chemicals, procedures, or equipment presenting unusual hazards inherent in their use have been used. Additionally, our dataset does not involve the participation of human or animal subjects.
10. **Use of patient images or case details**: Given that our dataset consists of images of Peruvian banknotes, it does not require the approval of ethics committees or informed consent from patients or volunteers.

We hope this ethical statement reinforces our commitment to integrity and ethics in research and ensures the quality and reliability of our dataset and its potential publication in Data in Brief.

## CRediT authorship contribution statement

**Nicolás Esleyder Caytuiro-Silva:** Conceptualization, Methodology, Software, Writing – original draft, Project administration. **Jackeline Melady Peña-Alejandro:** Investigation, Resources, Data curation, Conceptualization. **Eveling Gloria Castro-Gutierrez:** Conceptualization, Supervision, Project administration, Validation. **Jose Sulla-Torres:** Supervision, Validation, Resources. **Benjamin Maraza-Quispe:** Supervision, Validation, Resources.

## Data Availability

Peruvian Banknotes (Original data) (Mendeley Data)
